# Identification and functional characterization of regulatory variants in *DPP9* associated with COVID-19 severity

**DOI:** 10.1186/s13073-026-01703-0

**Published:** 2026-07-02

**Authors:** Gaëlle Farah, Magali Torres, Leo Henches, Hugues Aschard, Jade Ghosn, Xavier Duval, Milieu Intérieur Consortium, Laurent Abel, Andres Alcover, Philippe Bousso, Nollaig Bourke, Petter Brodin, Pierre Bruhns, Nadine Cerf-Bensussan, Ana Cumano, Christophe D’Enfert, Caroline Demangel, Ludovic Deriano, Marie-Agnès Dillies, James Di Santo, Gérard Eberl, Jost Enninga, Jacques Fellay, Ivo Gomperts-Boneca, Milena Hasan, Gunilla Karlsson Hedestam, Serge Hercberg, Molly A. Ingersoll, Olivier Lantz, Rose Anne Kenny, Mickaël Ménager, Hugo Mouquet, Cliona O’Farrelly, Etienne Patin, Antonio Rausell, Frédéric Rieux-Laucat, Lars Rogge, Magnus Fontes, Anavaj Sakuntabhai, Olivier Schwartz, Benno Schwikowski, Spencer Shorte, Frédéric Tangy, Antoine Toubert, Mathilde Touvier, Marie-Noëlle Ungeheuer, Christophe Zimmer, Matthew L. Albert, Darragh Duffy, Lluis Quintana-Murci, French COVID Cohort Study Group, Amal Abrous, Delphine Bachelet, Marie Bartoli, Lila Bouadma, Minerva Cervantes-Gonzalez, Anissa Chair, Charlotte Charpentier, Sandrine Couffin-Cadiergues, Nathalie De Castro, Diane Descamps, Hang Doan, Céline Dorival, Hélène Esperou, Aline-Marie Florence, François Goehringer, Maxime Goyat, Ikram Houas, Isabelle Hoffmann, Salma Jaafoura, Simon Jamard, Nadhem Lafhej, Cédric Laouénan, Soizic Le Mestre, France Mentré, Christelle Paul, Aurélie Papadopoulos, Marion Schneider, Coralie Tardivon, Sarah Tubiana, Aurélie Wiedemann, Pascal Rihet, Salvatore Spicuglia, Sandrine Marquet

**Affiliations:** 1 Aix-Marseille Univ, INSERM, TAGC, UMR1090, MarMaRa Institute, Marseille, France; 2 Equipe Labellisée Ligue Contre Le Cancer, Marseille, France; 3 Department of Computational Biology, Institut Pasteur, Université Paris Cité, 75015 Paris, France; 4 Department of Epidemiology, Harvard T.H. Chan School of Public Health, Boston, MA USA; 5 Université Paris Cité, Infection, Antimicrobials, Modelling, Evolution (IAME), INSERM, UMR1137, 75018 Paris, France; 6 Infectious and Tropical Diseases Department, Assistance Publique-Hôpitaux de Paris Nord, Bichat-Claude Bernard University Hospital, 75018 Paris, France; 7 Inserm CIC 1425, IAME, Bichat Claude Bernard Hospital, Paris, France; 8 CNRS, Marseille, France

**Keywords:** COVID-19, Severe disease, *DPP9*, Regulatory variants, Haplotype, Genetic association, Bioinformatics priorization, Functional validation, Transcription factors

## Abstract

**Background:**

Severe acute respiratory syndrome coronavirus 2 (SARS-CoV-2) infection leads to a wide-range of clinical outcomes, which have been extensively studied through genome-wide association studies (GWAS).

**Methods:**

Starting from lead genetic variants associated with COVID-19 infection and severity, we identified a subset of non-coding candidate variants with potential regulatory functions. We combined bioinformatics analysis and functional screening in three cell lines to provide evidence for regulatory activity. We then performed a genetic study to test the association of our selected candidates with disease susceptibility followed by the functional validation of the risk haplotype.

**Results:**

We prioritized two *DPP9* variants within a haplotype that increases the risk of severe COVID-19. This haplotype exhibited increased regulatory activity and altered transcription factor binding, suggesting its role in influencing COVID-19 severity by modulating DPP9 expression in immune and lung cell types.

**Conclusions:**

The interest of our study lies in the functional characterization of regulatory variants responsible for increased levels of DPP9 and lung damage observed in patients with severe COVID-19. These findings advance our understanding of genetic risk factors for COVID-19 and highlight functional SNPs that may guide future therapeutic research.

**Supplementary Information:**

The online version contains supplementary material available at https://doi.org/10.1186/s13073-026-01703-0.

## Background

The novel coronavirus SARS-CoV-2, responsible for COVID-19, was first reported in December 2019 and rapidly developed into a global pandemic. As of October 14, 2025, it has affected nearly a billion people and caused over 7.1 million deaths worldwide (WHO Coronavirus (COVID-19) Dashboard | WHO Coronavirus (COVID-19) Dashboard With Vaccination Data). The infection causes a wide spectrum of clinical outcomes, from asymptomatic carriers to severe respiratory distress and death. Epidemiological studies have identified several risk factors for severe COVID-19, including hypertension, diabetes, obesity, older age, coronary artery disease, and male sex [1]. In addition to these clinical risk factors, host genetic factors have emerged as significant contributors to COVID-19 susceptibility and severity.

The COVID Human Genetic Effort (CHGE) [2] identified several inborn errors of type I interferon (IFN) immunity associated with COVID-19 pneumonia in children under 16 years of age [3] and in patients younger than 60 [4]. They further demonstrated that rare loss-of-function variants in type I IFN-related genes can underlie life-threatening COVID-19 [5]. They suggested that insufficient type I IFN immunity in the respiratory tract during the early days of infection facilitates viral spread, leading to pulmonary and systemic inflammation [4]. Genome-wide association studies (GWAS) have also identified genetic loci associated with COVID-19 outcomes [6–8]. The COVID-19 Host Genetics Initiative (HGI) identified 13 loci associated with severe illness, hospitalization, and SARS-CoV-2 infection across multiple populations [9]. However, the functional implications of these variants remain poorly understood due to a lack of comprehensive experimental validation. Understanding their mechanistic roles is crucial, as they may affect gene expression, protein function and immune responses to SARS-CoV-2.

Genetic variations in the non-coding genome can disrupt the finely tuned control of gene expression, increasing disease risk or progression. Changes in cis-regulatory elements may alter gene regulation and make individuals more susceptible to certain conditions. However, identifying and characterizing these regulatory variants is technically challenging due to their abundance, cell- and tissue-type specificity, ability to regulate one or multiple genes over short or long genomic distances, and their complex modes of action [10]. Recent advances highlight the importance of identifying causal variants that contribute to the variability in COVID-19 clinical phenotypes [11–13]. This research seeks to deepen our understanding of the genetic underpinnings of COVID-19 and uncover novel insights into how these genetic variants may influence disease outcomes. To explore this, we focused on the 13 independent loci identified by the COVID-19 HGI GWAS [9]. Using bioinformatics and experimental approaches, we uncovered two variants that likely regulate the expression of the *DPP9* gene through epistatic interactions, providing new insights into the genetic determinants of COVID-19 susceptibility and severity.

## Methods

### Bioinformatic prioritization and functional annotation of genetic variants

Significant variants (*n* = 13) associated with different phenotypic outcomes of COVID-19 were identified as lead SNPs in the COVID-19 Host Genetics Initiative meta-analysis [9]. Using HaploReg v.4.1 (broadinstitute.org) and Ensembl 1000 Genome Phase 3 CEU, a list of variants of interest in LD with the 13 lead SNPs was created. HaploReg integrates regulatory genomic maps and data from ENCODE, GTEx, Roadmap Epigenomics, and the 1000 Genomes Project for the purpose of mining putative causal variants, cell types, regulators, and target genes for complex traits and diseases by calculating the LD between variants and assessing enrichment analysis [14]. For this analysis in a European population, with an r^2^ threshold > 0.6, we have identified 565 variants of interest. To prioritize those variants with potential functional effects, we performed a bioinformatic analysis using the Integrative Weighted (IW)-Scoring annotation tool (SNP Annotation Tool (snp-nexus.org)). This tool combines disease/phenotype and tissue/cell specificities with their related annotations into a single functional score since the tool evaluates eleven scoring methods derived from eight studies (CADD, FitCons, Eigen, FATHMM, GWAVA, DeepSEA, FunSeq2, ReMM) [15]. An integrative score is calculated for each variant as the linear weighted sum of the functional scores obtained using the different methods; this allowed us to rank the best candidates having the highest scores. That way, the final score is based on diverse genomic features regarding the context of surrounding sequence gene model annotations, evolutionary constraints, epigenetic measurements, functional predictions, and assays. Furthermore, the IW-scoring tool provides the K11 integrated score which incorporates all 11 functional prediction tools. We employed a two-tiered thresholding approach where we computed the non-parametric right tail probability with an empirical *P*-value as described by Wang et al [15]. Thus, we selected variants with scoring significance of *P* < 0.02 as well as a K11 score > 1.6. This resulted in 10 top-ranking SNPs after excluding two SNPs that showed no signs of functional characteristics according to the following approach. We checked if the SNPs were annotated as eQTLs using GTEx browser (GTEx Portal). Moreover, using ReMap 2022 (ReMap2022 (univ-amu.fr)), we checked TF/DNA binding based on ChIP-seq experiments. To confirm whether the SNPs resided in chromatin-accessible regions, ATAC-seq data was visualized using the UCSC Genome Browser (UCSC Genome Browser). This visualization assessed whether SNPs were located within regions marked by H3K27ac, open chromatin (ATAC-seq), or predicted regulatory activity (ENCODE).

### Functional screening of candidate SNPs

#### Promoter activity

To assess promoter activity, regions ranging from 504 to 769 bp, each containing one of the following variants (rs2277732, rs7255545, or rs55981795 for the reference allele), were cloned upstream of the *Luc2CP* gene into the *NheI-BglII* site of the plasmid pGL4.12 (E6671, Promega, Madison, WI, USA). The cloning was performed by GeneCust© (GeneCust, Boynes, France).

#### Enhancer activity

To assess enhancer activity, regions ranging from 453 to 875 bp, each containing one of the following variants (rs4801778, rs8080531, rs7255545, rs17206707, rs72711187, rs12482060, rs11088247, rs55981795, and rs75407602 for the reference allele) were cloned downstream of the *Luc2CP* gene into the *BamHI* site of the pGL4.12 plasmid containing the SV40 promoter. The cloning was performed by GeneCust© (GeneCust, Boynes, France). The pGL4.12-SV40 vector was constructed by Juliette Malfait by cloning the minimal SV40 promoter into the pGL4.12 vector (Promega, E6671) at the BglII and HindIII restriction sites.

### Bacterial transformation and midi prep

Lyophilized plasmids were resuspended in 10 µl MilliQ de-ionized nuclease-free water and were subsequently transformed into NEB® 10-beta electrocompetent *E. coli* DH10B strain (New England Biolabs, C3020K) by electroporation according to the manufacturer’s protocol. Transformed bacteria were cultured and harvested by centrifugation at 4000 rpm for 20 min, at 4⁰C, and plasmids were extracted using the QIAGEN Plasmid Plus Midi Kit (cat. nos. 12943 and 12,945; https://www.qiagen.com/us) following the manufacturer’s instructions.

### Cell culture

K562 cells (ATCC CLL-243), a chronic myelogenous leukemia cell line, and Jurkat cells (ATCC TIB-152), a T-cell acute lymphoblastic leukemia cell line, were cultured in Gibco RPMI 1640 medium (Thermo Fisher Scientific, Waltham, MA, USA), supplemented with 10% fetal bovine serum (FBS). A549 cells (ATCC CCL-185), a lung carcinoma cell line, were maintained as adherent monolayers in Dulbecco’s modified Eagle’s medium (DMEM/F12, Thermo Fisher Scientific, Waltham, MA, USA), supplemented with 10% FBS. All cell lines were grown at 37 °C in a humidified incubator with 5% CO_2_ and passed every 2–3 days at a density of 3 × 10^5^ cells per ml. Cultures were systematically tested and confirmed to be free from mycoplasma contamination.

### Gene reporter assay

For each assay, 10^6^ K562 or Jurkat cells were co-transfected with 200 ng of pRL-SV40 (a plasmid encoding Renilla luciferase) (Promega, E2231), and either 1µg of constructs of interest or 1 µg of control vectors using the Neon™ Transfection System (MPK5000, Thermo Fisher Scientific) according to the manufacturer’s instructions. A549 cells were co-transfected with 100 ng of pRL-SV40 (Promega, E2231), and either 1 µg of constructs of interest or 1 µg of control vectors using Lipofectamine™ 3000 Transfection Reagent kit (L3000008, Thermo Fisher Scientific). The negative control plasmid for promoter activity is an empty pGL4.12 basic and the negative control construct for enhancer activity is pGL4.12 with SV40 promoter. Sixteen hours after transfection, cells were split into two. Only half of the cells were stimulated with interferon alpha (IFNα) (SRP4594-100UG – Sigma-Aldrich) by adding one volume of media containing 100 ng/µl of IFNα for a final concentration of 100 ng/ml. Stimulation efficiency was validated using a pGL4.12-SV40 construct with the *OAS3* Epromoter cloned downstream of the *Luc2CP* gene. This construct was generated by Antoinette Van Ouwerkerk. Firefly and Renilla luciferase activities were measured 6 h after stimulation (24 h post-transfection), using the Dual-Luciferase® Reporter Assay System kit (E1980, Promega), according to the manufacturer’s protocol. Measurements were performed using 20 µl of cell lysates on the *VICTOR Nivo* machine (PerkinElmer). Firefly luciferase data were normalized to Renilla luciferase activity and expressed as fold change of relative light units over the respective negative controls.

### Cohorts and genetic analysis

Individual-level haplotype data came from two cohorts assembled as part of the EpiGenCOV consortium: hospitalized COVID-19 cases from the French COVID study and controls from the Milieu Interieur cohort. French COVID participants are critically ill cases of COVID-19 with COVID-19 infection confirmed by PCR and who either required respiratory support in hospital or died due to the disease. The patients were recruited across over 130 hospitals in France. The subset of participants included in the present analysis was recruited between February 2020 and November 2020. Milieu Interieur controls are individuals from the general population in France without known SARS-CoV-2 infection. The cohort includes over 1,000 healthy volunteers, stratified according to sex with a 1:1 ratio and age (5 decades of age, spreading from 20 to 69 years old) and recruited in France in 2012 and 2013. For the present analysis we used a subset of 457 French COVID cases and 982 controls from Milieu Interieur, who were all of European ancestry, and with both genome-wide genetic and covariates data (age, sex and body mass index).

Samples were genotyped with two different DNA arrays, and we imputed each cohort separately. Imputation was performed using the RICOPILI pipeline [16]. Prior to imputation, quality control procedures were applied to the genotyped data, excluding SNPs with call rates < 98%, minor allele frequency (MAF) < 1%, and Hardy–Weinberg equilibrium *P*-value > 1 × 10^–6^. Imputation was conducted using the 1000 Genome reference panel [17], which consists of 2,504 individuals at 84.7 million SNPs. Genotypes were phased using Eagle v2.3.5 [18] and imputed using minimac3 [19]. Imputation was done separately for cases and controls as the intersection of the genotyped SNPs between the two cohorts was small (123,309 variants). Imputation scores were 0.99 and 0.97 for rs2277732, and 0.88 and 0.92 for rs7255545 in French COVID and Milieu Interieur, respectively. Relatedness between samples and population stratification was derived using chip-wide SNP data passing stringent quality control. We extracted 2 haplotypes from the imputed data for each participant. We defined 4 types of haplotypes: G-C, G-A, A-C, and A-A, depending on the observed alleles for rs2277732 (first position) and rs7255545 (second position). All association models included the first 20 principal components of the genotype matrix, sex, sex squared, age, and age squared as covariates. Association test was performed using a linear regression between the genetic variants of interest (either a single SNP or haplotype) and COVID-19, using the following statistical model: $Y\sim {\widehat{\beta }}_{G}G+{\sum}_{i=1\dots n}{\widehat{\beta }}_{i}{C}_{i}$, where ${C}_{i}$ represents the covariate *i*. The joint test of rs2277732 and rs7255545 was performed using a multivariate Wald test of the regression coefficients ${\widehat{\beta }}_{G1}$ and ${\widehat{\beta }}_{G2}$ from the model: $Y\sim {\widehat{\beta }}_{G1}{G}_{1}+{\widehat{\beta }}_{G2}{G}_{2}+{\sum}_{i=1\dots n}{\widehat{\beta }}_{i}{C}_{i}$, where *G1* and *G2* represent rs2277732 and rs7255545, respectively.

The genotype of these two SNPs was successfully imputed in both cohorts, with imputation quality scores of 0.99 and 0.97 for rs2277732 in the case and control cohorts, respectively, and 0.88 and 0.92 for rs7255545 in the case and control cohorts, respectively.

### Site-directed mutagenesis

The Q5® Site-Directed Mutagenesis Kit (New England Biolabs, E0554S) was used to substitute the reference allele (C) of the cloned SNP rs2277732 with the alternative allele (A). Mutagenesis was performed by PCR using primers designed with NEBaseChanger (https://nebasechanger.neb.com/). The primer sequences are provided in Additional file 1: Table S1. PCR products were purified using the QIAquick® PCR Purification Kit (QIAGEN, 28,106) according to the manufacturer’s protocol. Mutations were validated by Sanger sequencing using primers specific to the region cloned upstream of the *Luc2CP* gene (Additional file 1: Table S1). Sequencing was performed by the Eurofins platform (Eurofins Genomics—Genomic services by experts) and chromatograms were analyzed with SnapGene Viewer (https://www.snapgene.com/).

## Gibson assembly

Plasmids with the reference and alternative alleles of rs2277732 were used to generate haplotype combinations (Alt/Alt, Ref/Ref and Ref/Alt) using the Gibson assembly method (Gibson Assembly® Master Mix – Assembly (E2611) NEB) following the manufacturer’s instructions. The fragment containing the reference allele (G) of rs7255545 was first retrieved by BamHI enzymatic digestion of the construct pGL4.12-SV40., purified on gel using the PureLink® Quick Gel Extraction Kit (Invitrogen, K2100-12) and cloned into the BamHI site downstream of the *Luc2CP* gene in the construct containing either allele of rs2277732. Cloning was validated by Sanger sequencing using primers that target the regions downstream of the Luc2CP gene (Additional file 1: Table S1). Using the Ref-Ref and Alt-Ref constructs, we generated the Ref-Alt and Alt-Alt constructs using the Q5 site-directed mutagenesis with the following primers targeting rs7255545 (Additional file 1: Table S1). Mutations were validated by Sanger sequencing with the same primers used to validate Gibson cloning. The resulting constructs were amplified and transfected into K562, Jurkat, and A549 cells as described previously. Luciferase assays were performed as described above three times in triplicate.

### Haplotype-specific gene reporter assay

In order to assess the allelic effect of the SNPs on gene regulation, haplotype-specific gene reporter assays were carried out in A549, K562 and Jurkat cells without stimulation and in A549 and K562 in different conditions of stimulation. The cells were transfected with the plasmids as previously described. Cells were split 4 h after transfection for stimulation. i) Interferon alpha (IFNα) stimulation: one volume of media containing 100 ng/µl of IFNα (SRP4594-100UG – Sigma-Aldrich Chimie) was added to the cells for a final concentration of 100 ng/ml during 24 h; ii) Interferon gamma (IFNγ) stimulation: one volume of media containing 100 ng/µl of IFNγ−1b (130–096–484, Miltenyi Biotec) was added to the cells for a final concentration of 100 ng/ml during 24 h; iii) Tumor necrosis factor alpha (TNFα) stimulation: one volume of media containing 10 ng/ml of TNFα (ab9642, Abcam) was added to the cells for a final concentration of 10 pg/ml during 24 h. SARS-CoV-2 protein stimulation was carried out on A549 cells only by adding the recombinant proteins 4 h after transfection as follows: i) 10 µl of recombinant human coronavirus SARS-CoV-2 Spike Glycoprotein RBD (His tag) (Spike) (ab275986-50ug, Abcam) were added to the cells for a final concentration of 50 ng/µl for 24 h; ii) 5 µl of recombinant human coronavirus SARS-CoV-2 Nucleocapsid protein (His tag) (Nucleocapsid) (ab273530-100ug, Abcam) were added to the cells for a final concentration of 50 ng/µl for 24 h; iii) 10 µl of Spike and 5 µl of Nucleocapsid were added to the cells for a final concentration of 50 ng/µl each for 24 h.

### Gene expression analysis

Exponentially growing A549 and K562 cells were stimulated for 6, 12, 24 and 48 h using IFNα, IFNγ, TNFα, Spike, Nucleocapsid and a mix of Spike and Nucleocapsid as previously described. RNA was extracted using RNeasy Plus Mini Kit (Qiagen 74,106) according to the manufacturer’s instructions. 1 µg of total RNA was reverse transcribed using LunaScript RT SuperMix Kit (New England Biolabs, E3010L). qPCR reactions were made using SYBR green Master Mix (Thermo Fisher Scientific, 4,367,660) and measurement was made using the Applied Biosystems Quant Studio 6 Flex Real Time PCR system. 25 ng cDNA was used for the PCR and relative expression was analyzed using the comparative delta CT method. The *GAPDH* gene was used to normalize samples. Each point in the figures corresponds to the means of independent RNA and cDNA preparation. Values were normalized to unstimulated cells. The primers used are detailed in Additional file 1: Table S1.

### Nuclear extracts preparation and electrophoretic mobility shift assay (EMSA)

Nuclear proteins were extracted from the A549, K562 and Jurkat cell lines using the Nuclear extract kit (Active Motif, Cat#40,010) according to the manufacturer’s instructions. For the preparation of nuclear extracts, 9 × 10^6^ fresh wild-type cells were used. Protein concentrations were determined by a Bradford protein assay (Bio-Rad). EMSA was performed using the Gelshift Chemiluminescent EMSA Kit (Active Motif, Cat#37,341) according to manufacturer’s instructions. For the oligo probe, complementary biotinylated and non-biotinylated single-stranded 30-bp oligonucleotides containing each allele of the SNPs rs2277732 and rs7255545 (sequences in Additional file 1: Table S1) were annealed. For competition experiments, a 50-fold molar excess of unlabeled probe were added. For each reaction, we have used 10 µg of nuclear extract, 2 µl of 0.2 µM of biotinylated oligo duplexes, and 2 µl of 10 µM of non-biotinylated competitor oligo duplexes. Complexes were separated on 6% polyacrylamide Novex DNA Retardation Gels prepared with 0.5X TBE gel buffer. Images were captured on the iBright 750 (Invitrogen).

### Transcription Factor motif analysis

The impact of the selected variants on transcription factor binding sites (TFBS) was evaluated using the RSAT tool [20], JASPAR database [21] and FABIAN-variant tool [22]. Briefly, RSAT uses Position-Specific Scoring Matrices (PSSMs) to evaluate the affinity of a given TF to a given sequence with each variant. RSAT calculates the probability that the tested sequence binds to a TF, and the ratio of variant *P*-values. Variant sequences were scanned with position-specific scoring matrices (PSSM) from the JASPAR database. FABIAN-variant estimates changes in binding affinity by assessing the gain or loss of factor motifs at SNP-affected sites. For rs2277732 and rs7255545, we evaluated their predicted impact on the associated TFBSs using Jaspar2022.

### Statistical tests

Graphs were generated using GraphPad Prism 10.1.12 and Rstudio 4.4.0. Data analysis was conducted using GraphPad Prism and RStudio. Data were presented as mean ± standard deviation (SD). A one-tailed Student t test and two-tailed Wilcoxon-Mann–Whitney test were used to evaluate the statistical significance of promoter or enhancer activity of candidate regions tested in the gene reporter assays. Kruskal–Wallis and post-hoc Wilcoxon-Mann–Whitney analysis were applied to evaluate the statistical significance of allele-specific haplotype under different conditions of stimulation. Two-way ANOVA was used to test for interaction between the stimulations and the two different haplotypes. Luciferase values were normalized to Renilla and fold-change was expressed relative to controls. A two-tailed unpaired Student t test was performed to evaluate the statistical significance of qPCR results between two groups. Statistical significance was determined by the *P*-values as follows: * *P* < 0.05, ** P < 0.01, *** P < 0.001, **** P < 0.0001. Details of each statistical test are indicated in the figure legends. Exact *P*-values of statistical tests are provided in the Source Data file

## Results

### Prioritization of regulatory variants associated with COVID-19

**Fig. 1:**
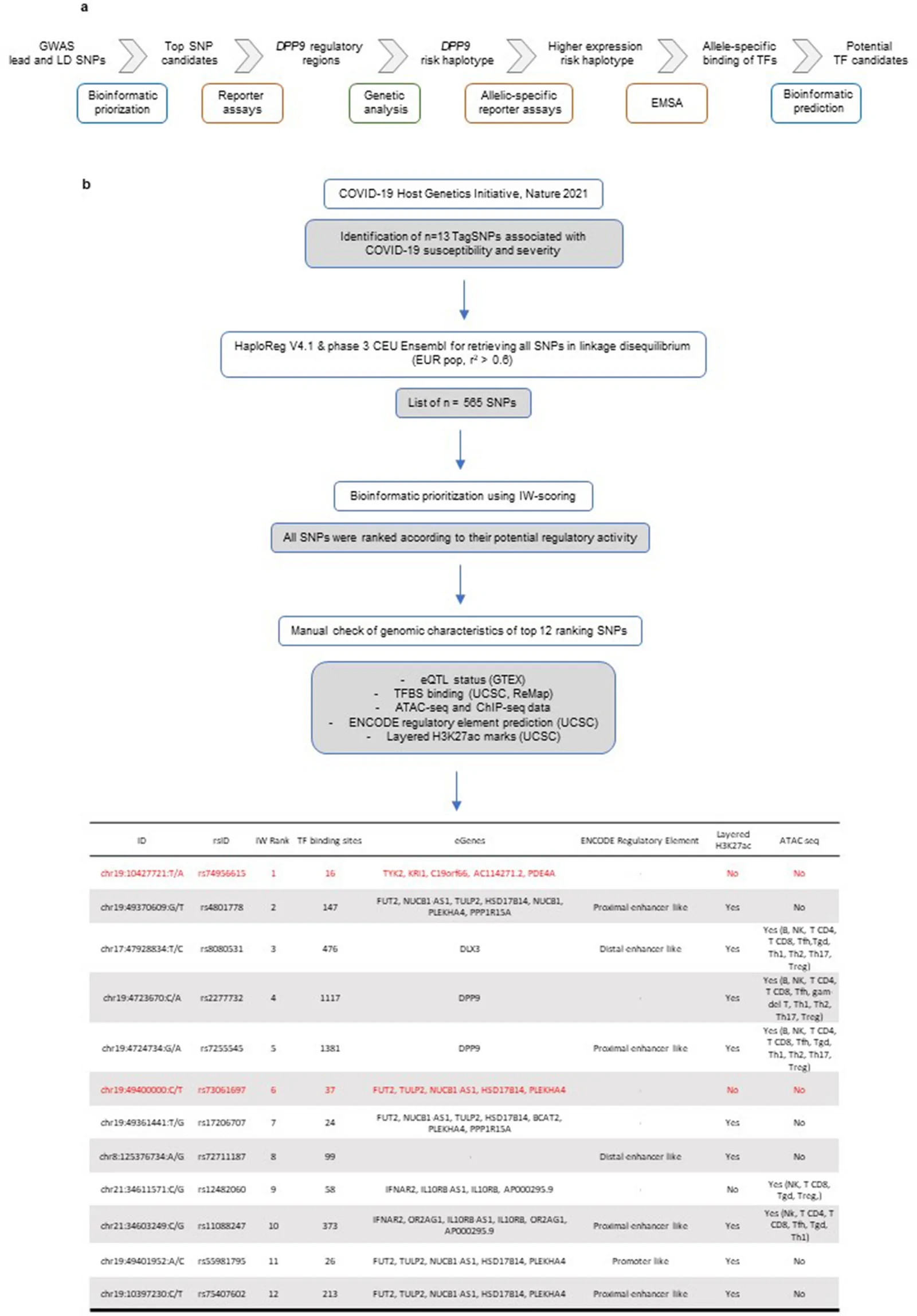
Study design and bioinformatics pipeline for prioritizing candidate causal variants at COVID-19 associated loci. **a** Schematic overview of the study indicating the main methodological steps and results. **b** From the COVID-19 HGI meta-analysis identifying 13 lead SNPs associated with COVID-19 susceptibility and severity, SNPs in linkage disequilibrium (LD) with an r^2^ threshold of 0.6 in a European population were retrieved, resulting in a list of 565 SNPs. These SNPs were prioritized using the IW-scoring tool. The top 12 ranking SNPs were further examined for key genomic characteristics, including TF binding sites, target eGenes, overlap with ENCODE regulatory element predictions, H3K27ac histone modification marks, and ATAC-seq peaks. A table is provided summarizing these characteristics for each SNP. SNPs selected for downstream validation are indicated in black, while SNPs excluded from validation are highlighted in red

We focused our analysis on the 13 lead variants showing genome-wide-significant associations (with the threshold of *P* adjusted < 1.67 × 10^–8^) with at least one COVID-19 phenotype (SARS-CoV-2 infection, hospitalization, or critical illness) (Additional file 1: Table S2), identified by the COVID-19 Host Genetics Initiative, across 49,562 European patients from 19 countries^9^. We identified 565 SNPs in linkage disequilibrium (LD) with these lead SNPs in the European population with an r^2^ threshold of 0.6 using HaploReg V4.1 [14] and Ensembl 1000 Genome Phase 3 CEU (Fig. 1). We prioritized those variants based on their potential regulatory effect using the Integrative Weighted (IW)-scoring tool [15], with detailed rankings provided in Additional file 1: Table S3. To ensure strong evidence of regulatory function and causality, we evaluated whether the SNPs from the IW-scoring prioritized list were annotated as expression quantitative trait loci (eQTL) using the GTEx browser [23] and assessed their colocalization with ATAC-seq peaks, H3K27ac ChIP-seq peaks, and transcription factor binding sites (TFBS) using ReMap 2022 [24] (Fig. 1). ENCODE [25] data were used to confirm whether these SNPs were located in promoter or enhancer-like regions. Based on this thorough curation, we excluded two of the top-ranking SNPs rs74956615 and rs7306169, as they showed no evidence of regulatory activity (Fig. 1). Finally, we selected for experimental validation the top 10 candidates showing a K11 IW-score above 1.6 and a scoring significance of *P* < 0.02 (Additional file 1: Table S3), based on the distribution of all variant scores as described by Wang et al [26].

### Assessment of regulatory activity of candidate SNP-containing regions

**Fig. 2:**
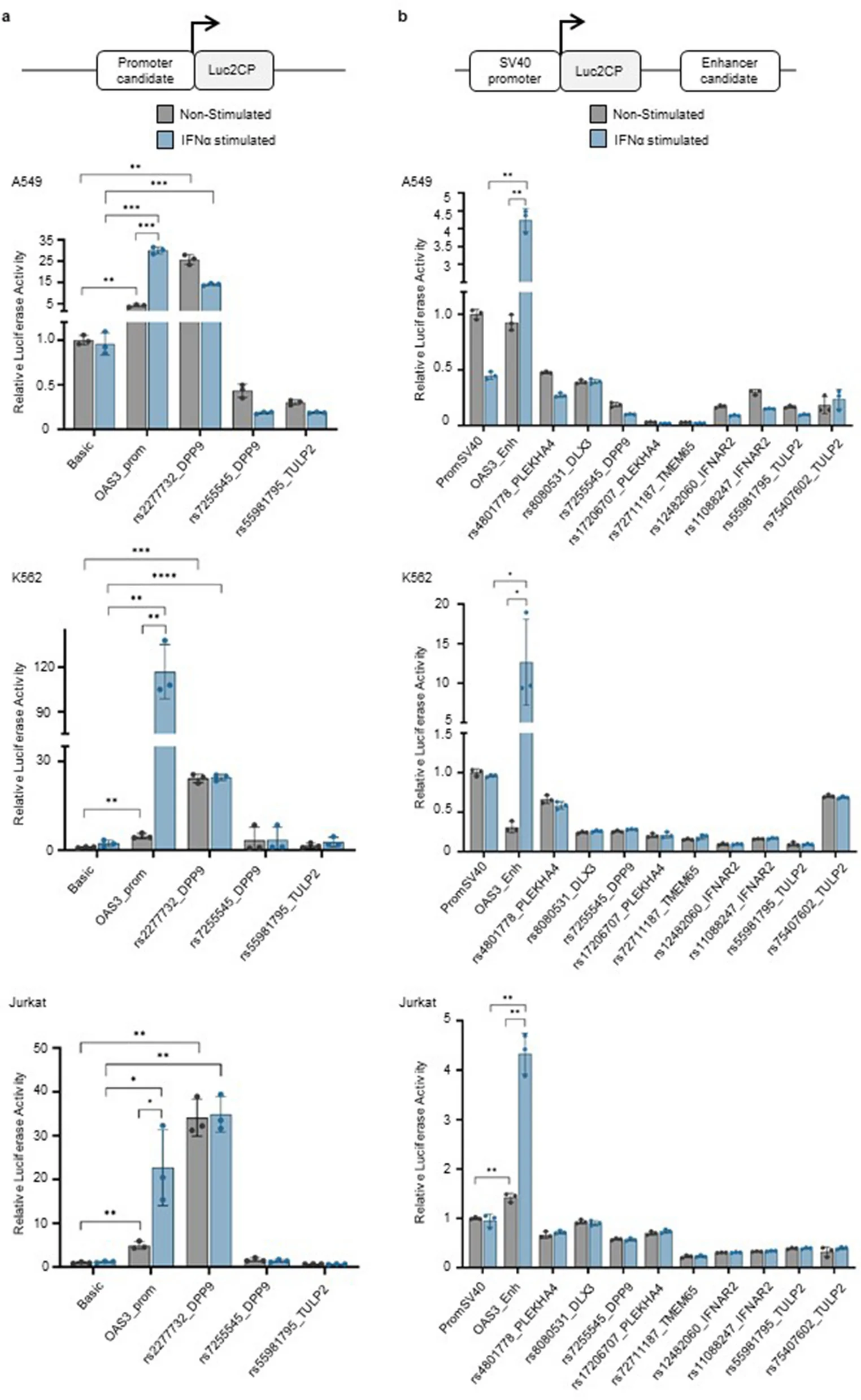
Functional screening of candidate regulatory variants across different cell lines. **a**,** b** Fold-change induction of luciferase activity is shown for regions containing the 10 selected candidate SNPs in A549, K562, and Jurkat cell lines. **a** Relative luciferase activity of three regions, each containing one of the following SNPs: rs2277732, rs7255545, and rs55981795, cloned as promoters upstream of the *Luc2CP* gene, and expressed as fold change relative to the basic plasmid. **b** Relative luciferase activity of nine regions, each containing one of the following SNPs: rs4801778, rs8080531, rs7255545, rs17206707, rs72711187, rs12482060, rs11088247, rs55981795 and rs75407602, cloned as enhancers downstream of the *Luc2CP* gene and containing the minimal SV40 promoter, expressed as fold change relative to the PromSV40 plasmid. Firefly luciferase activity was measured 24 h post-transfection, without stimulation (grey bar) or following interferon-alpha (IFNα) stimulation for 6 h (blue bar). The IFNα-inducible region of OAS3 was cloned (**a**) upstream of the luciferase gene or (**b**) downstream and served as a positive control for stimulation. Results were normalized to Renilla luciferase activity. Data (bar plot) mean ± SD of n = 3 of 3 independent experiments (dots). Significance was determined by a one-tailed Student t test and *p*-values are represented as asterisks (* *P* < 0.05, ** *P* < 0.01, *** *P* < 0.001, **** *P* < 0.0001). Source data and exact *P*-values of statistical tests are provided in the Source Data file

Based on genomic characteristics (distance from the TSS, annotation from ENCODE), we tested ten loci containing candidates SNPs. One region (rs2277732_*DPP9*) was analyzed as a promoter, seven as potential enhancers (rs4801778_*PLEKHA4*, rs8080531_*DLX3*, rs17206707_*PLEKHA4*, rs72711187_*TMEM65*, rs12482060_*IFNAR2*, rs11088247_*IFNAR2*, rs75407602_*TULP2*) and two for both promoter and enhancer activity (rs7255545_*DPP9*, rs55981795_*TULP2*) (Fig. 2a and 2b). For each variant, we selected a region between 453–873 bp centered on the TF density peak identified by ReMap 2022 (Additional file 1: Table S4). Indeed, to preserve the activity of the candidate genomic regions containing the prioritized SNPs, we selected the regions overlapping the corresponding ReMap density peak (Additional file 1: Table S4), thereby ensuring the inclusion of TF binding sites identified by ChIP-seq experiments.

Gene reporter assays were performed in two immune cell lines (K562, Jurkat) and one lung epithelial cell line (A549) with or without interferon-alpha (IFNα) stimulation to mimic a viral infection [26] (Fig. 2a and 2b). These models were chosen because immune and lung cells are central to SARS-CoV-2 pathogenesis [27]. Cells were transfected with constructs to test promoter or enhancer activity. As a positive control of IFNα induction, we used an *OAS3* inducible regulatory region with both promoter and enhancer activities [28] (Fig. 2).

As shown in Fig. 2a, the region rs2277732_*DPP9* containing the reference allele C of rs2277732 showed strong promoter activity in all three cell lines. IFNα stimulation had no effect on the tested regions in Jurkat and K562, but reduced activity in the A549 cells. No enhancer activity was detected for any region relative to the SV40 promoter construct (Fig. 2b).

### Genetic and genomic characterization of the *DPP9* regulatory locus

**Fig. 3:**
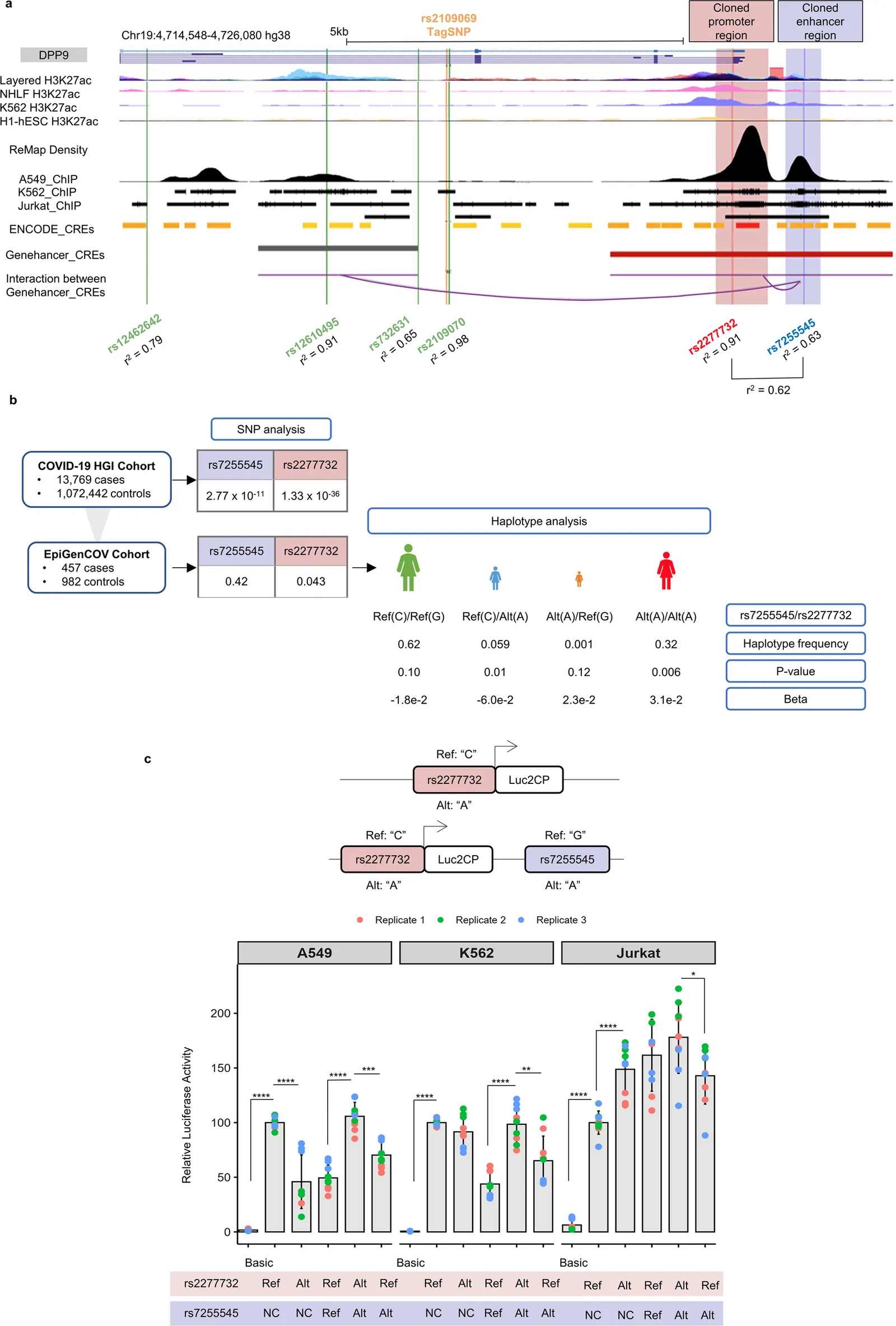
Severe COVID-19 risk haplotype in *DPP9* drives elevated gene expression. **a** Genomic characterization of the *DPP9* regulatory locus. A UCSC Genome Browser screenshot shows the genomic region surrounding the *DPP9 gene*. The lead SNP rs2109069 is marked in orange, while SNPs in linkage disequilibrium (LD) are in green, red and blue. Our two candidate regions containing rs7255545 and rs2277732 are highlighted in blue and red, respectively. The r^2^ values for LD between rs2109069 and each SNP, as well as between the two candidate SNPs, are indicated. Regulatory features include H3K27ac histone marks across various cell types (NHLF, K562 and H1-hESC), transcription factor ChIP-seq peaks (ReMap density), and TF ChIP-seq data for A549, K562 and Jurkat cells. ENCODE and Genehancer predicted CREs, and their interactions are also shown. **b** Genetic association of rs7255545 and rs2277732 with COVID-19 critical illness. Imputation data from the COVID-19 HGI and the EpiGenCOV cohorts show *P*-values and $\widehat{\beta }$ for the risk associated alternative (A) alleles. Haplotype analysis in EpiGenCOV reveals four haplotypes (Ref-Ref, Ref-Alt, Alt-Ref, Alt-Alt), with their respective frequencies, *P*-values, and $\widehat{\beta }$ supporting association with critical illness. **c** Luciferase reporter assays of recombinant haplotypes. Constructs used for functional validation are shown. The region containing each allele of rs2277732 (red) was cloned upstream of the *Luc2CP* gene (Ref-NC or Alt-NC), alone or with the region containing each allele of rs7255545 (blue) downstream. Constructs were tested three times in triplicate (as indicated by the colored dots) in A549, K562, and Jurkat cells. Results are shown as relative luciferase activity versus Ref-NC. Bars represent mean ± SD of n = 9 experiments (dots) represent 3 independent replicates with 3 independent transfections per replicate. Significance was determined using a two-tailed Wilcoxon-Mann–Whitney test and *P*-values are indicated as asterisks (* *P* < 0.05, ** *P* < 0.01, *** *P* < 0.001, **** *P* < 0.0001). Source data and exact *P*-values of statistical tests are provided in the Source Data file.

Based on gene reporter assay results, we focused on *DPP9*, located near rs2277732, for further analysis. As shown in Fig. 3a, the lead SNP rs2109069, previously associated with all three COVID-19 outcomes (severe illness, hospitalization, and SARS-CoV-2 infection) [9], showed no overlap with H3K27ac mark or ChIP-seq peaks according to ReMap, suggesting it has no functional role. This is consistent with its low IW-score and its ranked position 360.

In the European population, rs2109069 is in LD (r^2^ > 0.6) with six SNPs (Fig. 3a). Among them, rs2277732 and rs7255545 had high IW scores, ranked 4th and 5th, respectively (Additional file 1: Table S3) and showed strong regulatory potential. ENCODE data revealed that both SNPs lie in active regulatory regions, with strong H3K27ac and ATAC-seq signals in Human lung fibroblasts (NHLF) and erythro-myeloid cells (K562) (Fig. 3a). They also overlap with ATAC-seq peaks and multiple transcription factor binding sites in A549, K562, and Jurkat cells, the three cell lines used for the functional screening.

According to ENCODE, rs7255545 is in an enhancer region that may interact with the rs2277732 promoter region (Fig. 3a). The RegulomeDB database provides additional evidence supporting the functional relevance of rs2277732 and rs7255545, assigning them the strongest prediction rank and score values of 0.98 and 0.55, respectively. Both SNPs act as eQTLs for *DPP9*, suggesting they may jointly regulate its expression.

### Association of *DPP9* regulatory variants with COVID-19

We first examined the association of rs2277732 and rs7255545 SNPs with COVID-19 phenotypes (susceptibility, hospitalized and critically ill) in individuals of European ancestry. The association results were pulled from the summary statistics generated by the COVID-19 Host Genetics Initiative (COVID-19 HGI) available at https://www.covid19hg.org/. Strong associations were found between rs2277732 ($\widehat{\beta }$ = 0.144, *P* = 5.82 × 10^–38^) and rs7255545 ($\widehat{\beta }$ = 0.084, *P* = 2.77 × 10^–11^) and the critical illness status, indicating that the alternative A allele of each SNP is the risk allele (Fig. 3b). We also tested the association of these two SNPs in samples from EpiGenCOV, a French consortium including hospitalized COVID-19 cases from French COVID and individuals from the general population from the Milieu Intérieur cohorts [29]. We observed a nominal association with rs2277732 ($\widehat{\beta }$ = 0.023, *P* = 0.043), but no significant association with rs7255545 ($\widehat{\beta }$ = 0.0092, *P* = 0.42), in this sub-sample of limited size, when including only one variant in the model (Fig. 3b). Interestingly, however, the joint model including the two variants produced a substantially stronger signal (*P* = 0.0047), and opposite regression coefficients (${\widehat{\beta }}_{rs2277732}$ = 0.086 and ${\widehat{\beta }}_{rs7255545}$ = −0.070), suggesting that, taken together, the two variants capture a more complex structure than the sum of their individual effects.

Given the genomic proximity of SNPs rs7255545 and rs2277732, a haplotype analysis was performed to assess the combined effect of the two *DPP9* SNPs on COVID-19 critical illness. Four haplotypes were detected in the studied population, with varying numbers of individuals: ((1) *rs*7255545 **G**—rs2277732 **C**: n = 1793; (2) rs7255545 **G**—rs2277732 **A:** n = 148**;** (3) rs7255545 **A**—rs2277732 **C**: n = 4; (4) rs7255545 **A**—rs2277732 **A**: n = 933) (Fig. 3b). The frequency of the A-A haplotype, consisting of the alternative alleles of the two SNPs, was higher in the French COVID cohort than in the Milieu Intérieur control group. The A-A haplotype was significantly associated with a higher risk of severe COVID-19 compared to the general population (*P* = 0.006, $\widehat{\beta }$ =0.031) (Fig. 3b). We also explored a homozygote model and obtained similar results (*P* = 0.005, $\widehat{\beta }$ = 0.068). As for the joint analysis, the haplotype analysis strengthened the association with severe COVID-19, as compared to individual SNP analyses. This is supported by promoter-enhancer interaction data from the GeneHancer database [30], which demonstrates interaction between the two regulatory elements containing each of these SNPs (Fig. 3a). This haplotype analysis could not be carried out in the larger cohort of COVID-19 HGI subjects, as individual data for these two SNPs, required for phasing and haplotype reconstruction, were not available.

### Higher regulatory activity linked to the *DPP9* risk haplotype

To evaluate the regulatory effect of rs2277732 alleles alone or in combination with rs7255545 alleles, we performed allele-specific gene reporter assays. First, the 769 bp region containing the rs2277732 SNP was cloned upstream of luciferase with either the reference C (Ref) or alternative A (Alt) allele. Subsequently, we added the 524 bp region containing rs7255545 downstream of luciferase, as ENCODE data suggests it may act as a proximal enhancer-like region (Fig. 1). The reference G (Ref) and alternative A (Alt) alleles of rs7255545 were cloned with the C (Ref) and A (Alt) alleles of rs2277732, respectively, to match the two most frequent haplotypes in our study population, with frequencies of 0.62 and 0.32 (Fig. 3c). We also tested the construct with the A (Alt) allele of rs7255545 combined with the C (Ref) allele of rs2277732, which has a frequency of 0.059 in the cohort. However, we did not assess the construct with the G (Ref) allele of rs7255545 combined with the A (Ref) allele of rs2277732 due to their very low frequency (0.001). Gene reporter assays were performed in A549, K562, and Jurkat cell lines. Consistent with our previous screening, the region containing rs2277732 showed strong promoter activity all cell lines (*P* < 10^–4^) (Fig. 3c). Specifically, the C reference allele exhibited significantly stronger activity than the A allele in A549 cells (*P* = 4.11 × 10⁻^5^), while the A allele was associated with higher luciferase activity in Jurkat cells (*P* = 8.22 × 10⁻^5^, Fig. 3c). When both SNPs were tested together, the A–A risk haplotype (alternative alleles) showed the strongest luciferase activity about 2.1 and 2.2-fold higher than the non-risk C–G haplotype (reference alleles) in both the A549 lung cell line and K562 cells, respectively (*P* = 4.11 × 10⁻^5^). The protective C-A haplotype, consisting of the C reference allele of rs2277732 and the A alternative allele of rs7255545, showed reduced activity than the risk haplotype across all three cell lines (A549, *P* = 1.6 × 10^–4^, K562, *P* = 5.67 × 10^–3^, and Jurkat, *P* = 1.87 × 10^–2^).

These findings suggest that the combined effect of these SNPs, rather than a single causal variant at the *DPP9* locus, may be responsible for the association with severe COVID-19. The consistent association of the risk haplotype with the highest luciferase activity across cell lines supports the hypothesis that these SNPs are functional variants, with their combined effect likely contributing to susceptibility to severe COVID-19, as indicated by our genetic data.

### Expression of *DPP9* varies depending on different stimuli

**Fig. 4:**
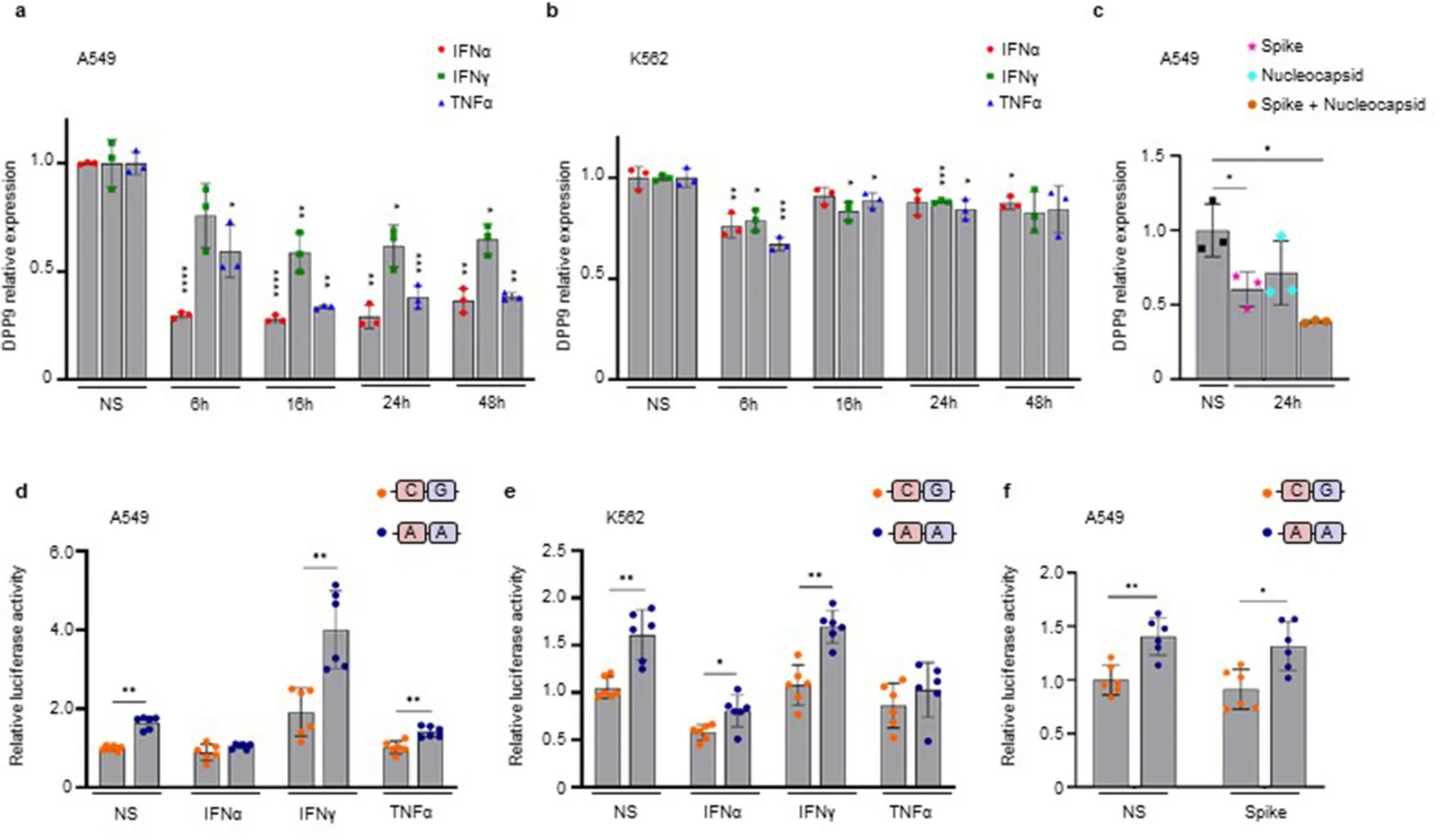
*DPP9* gene expression and regulation are affected by viral infection, represented by various stimulations.** a** mRNA analysis of *DPP9* in A549 with or without cytokine stimulations. **b** mRNA analysis of *DPP9* in K562 with or without cytokine stimulations. The cells were stimulated with different molecules mimicking viral infection, including interferon alpha (IFNα), interferon gamma (IFNγ) and tumor necrosis factor alpha (TNFα). *DPP9* expression was measured by RT-qPCR at 6-, 16-, 24-, and 48-h post-stimulation in A549 (a) and K562 cells (b) and compared to non-stimulated (NS) cells. **c** *DPP9* mRNA levels in A549 cells after 24-hours of stimulation with recombinant human coronavirus SARS-CoV-2 Spike glycoprotein RBD (Spike) and recombinant human coronavirus SARS-CoV-2 Nucleocapsid protein (Nucleocapsid) or both. **d-f** Luciferase reporter assays evaluating regulatory activity of non-risk (Ref/Ref; orange dots) and risk (Alt/Alt; blue dots) haplotypes in A549 (d, f), and K562 (e) under cytokine (d, e) or Spike (f) stimulations for 24-hours. Cytokine stimulations were performed using IFNα, IFNγ and TNFα. Data (bar plot) shows DPP9 relative expression normalized to *GAPDH* as mean values ± SD of *n* = 3 independent replicates (dots) (**a, b, c**). Significance was determined by a two-tailed Student t-test and *P*-values are indicated as asterisks (* *P* < 0.05; ** *P* < 0.01; *** *P* < 0.0001; **** *P* < 0.00001) (**a, b, c**). Luciferase data (bar plot) show fold change induction of luciferase activity represented as mean ± SD of n = 6 independent replicates (dots). Significance was determined by Kruskal–Wallis with post-hoc Wilcoxon-Mann–Whitney analysis as well as two-way ANOVA for interaction. *P*-values are represented as asterisks (* *P* < 0.05; ** *P* < 0.01). Source data and exact *P*-values of statistical tests are provided in the Source Data file

We assessed whether *DPP9* expression was influenced by cytokine stimulation in A549 (Fig. 4a) and K562 cells (Fig. 4b), which yielded the most convincing results in the luciferase assays. *DPP9* mRNA levels were measured at several time points, both without and with stimulation by IFNα, IFNγ, and TNFα. In A549 cells, *DPP9* expression significantly decreased under all cytokine treatments and at all time points. In K562 cells, although significant, the decrease in *DPP9* expression was weaker and observed at most, but not at all, time points following stimulation. To verify the effectiveness of the cytokine treatments, mRNA levels of inducible control genes were analyzed (Additional file 2: Fig S1). Together, these findings indicate that *DPP9* expression is more strongly affected by cytokine stimulation in A549 cells than in K562 cells.

We then tested *DPP9* expression in A549 cells stimulated with recombinant human coronavirus SARS-CoV-2 Spike Glycoprotein RBD (Spike) and recombinant human coronavirus SARS-CoV-2 Nucleocapsid protein (Nucleocapsid) (Fig. 4c). The efficiency of these recombinant proteins was previously demonstrated [31–35]. *DPP9* expression decreased significantly upon stimulation with Spike alone and with the combination of Spike and Nucleocapsid, with no significant difference between the two conditions. Since the Spike protein is located on the viral surface and binds to the ACE2 receptor expressed on epithelial cells, including A549, subsequent experiments were performed using only Spike for validation.

### Increased regulatory activity of the *DPP9* risk haplotype under stimulation

Next, we asked whether various stimulation affected the regulatory activity of the two *DPP9* haplotypes using a reporter assay. Kruskal–Wallis tests showed significant differences between haplotypes under cytokine stimulations in A549 and K562 cells (*P* < 1 × 10^–4^). In A549 cells, the risk haplotype consistently displayed significantly higher enhancer activity than the non-risk haplotype following IFNγ and TNFα stimulation, similar to non-stimulated conditions (Fig. 4d). Notably, there was a significant interaction between haplotype and stimulation effects. Although the risk haplotype induced greater luciferase activity than the non-risk haplotype under both conditions, this difference was markedly amplified under IFNγ stimulation (*P* = 0.007) compared with non-stimulated cells. In K562 cells, the risk haplotype also exhibited higher activity under IFNα and IFNγ stimulation (Fig. 4e), although no interaction effect was detected.

We further evaluated the effect of Spike stimulation in A549 cells (Fig. 4f). In line with previous observations, the risk haplotype showed higher regulatory activity than the non-risk haplotype (*P* = 3.4 × 10^–3^), with differences comparable to those observed in non-stimulated conditions, suggesting that Spike does not alter haplotype-dependent regulation.

### The two *DPP9* regulatory variants impact the binding of transcription factors

**Fig. 5:**
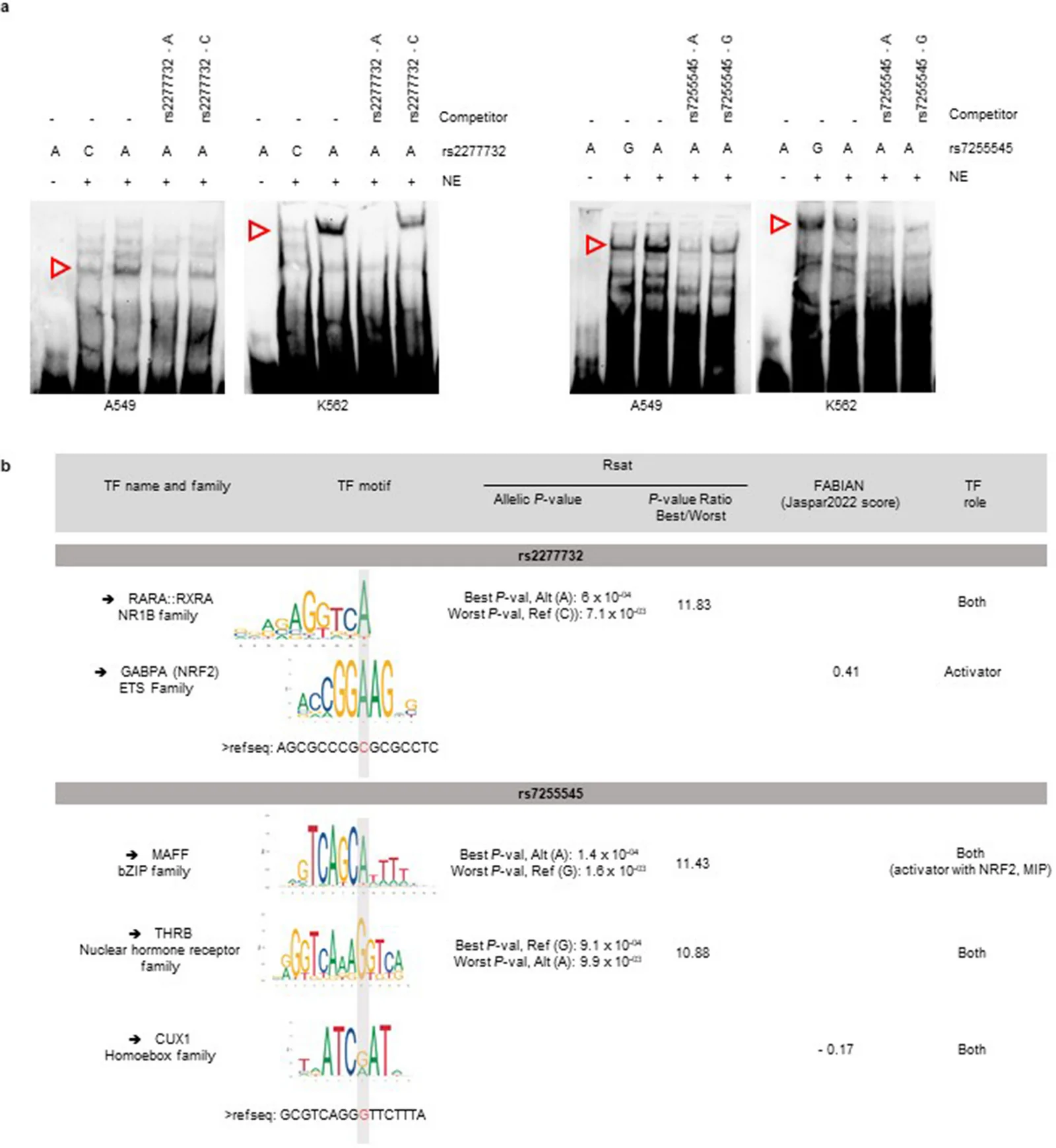
*DPP9* variants alter the binding of TFs. **a** EMSAs were performed to assess the effect of rs2277732 and rs7255545 alleles on TF binding in A549 and K562 cell lines, using corresponding biotinylated probes. Binding specificity was evaluated through competition assays with a 50-fold excess of unlabeled probes for each allele and SNP. EMSA experiments were independently repeated twice, yielding consistent results. **b** In silico analysis using RSAT and FABIAN-variant analyses were conducted to evaluate the impact of the allelic configuration of rs2277732 (top) and rs7255545 (bottom) on transcription factor (TF) binding site (TFBS) motifs. The figure presents the predicted TF, their families, TF motifs alignment to the reference genomic sequence with each SNP position in red. The analysis reports the *P*-value of TF fixation for each allele for both SNPs, as well as the *P*-value ratio (as computed by RSAT), reflecting potential disruption of TF binding due to the allelic variation and the Jaspar2022 scores provided by FABIAN-variant. The potential role of each TF is also provided

To validate allele-specific protein binding, we performed electrophoretic mobility shift assay (EMSA) using 30-bp biotinylated probes for rs7255545 and rs2277732 SNPs and nuclear extracts from the three cell lines (Fig. 5a). Both SNPs exhibited allele-imbalanced binding for nuclear extract (NE) from A549 and K562 cells (Fig. 5a). For rs2277732, the affinity of the complex to the risk-A allele probe was higher in comparison to the C allele probe in presence of NE from both cell lines (Fig. 5a). For rs7255545, higher affinity was observed with the risk-A allele probe in A549 whereas the affinity was lower with the risk-A allele probe in K562 cells (Fig. 5a). Competition of biotinylated A allele of rs2277732 with excess of non-biotinylated A allele more efficiently competed away allele-specific bands than excess of non-biotinylated C allele, demonstrating specificity of the protein-DNA complexes from A549 and K562 (Fig. 5a).

The risk rs7255545 A allele displayed stronger competition from unlabeled A allele probe as compared to G allele probe in A549. In K562, the complex was strongly reduced in the presence of an excess of unlabeled oligonucleotides containing the G allele of rs7255545 (Fig. 5a). No allele-specific binding was observed in the Jurkat cells (Additional file 2: Fig S2). These results indicate that rs2277732 and rs7255545 directly affect nuclear factors binding in a cell type-specific manner, likely regulating *DPP9* expression in lung and myeloid cells.

### Identification of potential transcription factors

To understand how rs2277732 and rs7255545 affect allele-specific gene regulation, we investigated their effects on transcription factor binding sites (TFBS) in silico using RSAT [20] and FABIAN-variant [22] tools. This bioinformatics analysis revealed several TF binding sites overlapping the two SNPs. We focused on TFs for which preferential binding to a specific allele was consistent with our EMSA assays. Based on RSAT, RARA::RXRA and CTCFL, showed preferential binding to the alternative (A) allele of rs2277732 (Fig. 5b, Additional file 1: Table S5). For rs7255545, MAFF showed increased binding to the alternative (A) allele, while PAX3 and THRB exhibited preferential binding to the reference (G) allele (Fig. 5b, Additional file 1: Table S5). Considering their dual roles as activators or repressors and the higher expression of the risk haplotype (Alt/Alt), RARA::RXRA, MAFF and THRB emerged as top candidates (Fig. 5b).

FABIAN-variant predicted TFBS gain and losses at both SNPs, some of which have been experimentally validated by ChIP-seq (Additional file 1: Table S6). GABPA also showed ChIP-seq evidence at rs2277732 in K562 and A549 cells according to RegulomeDB. For rs7255545, CUX1 binding was lost with the A allele. This TF may act as a repressor (Fig. 5b), shows evidence of ChIP-seq in K562 (RegulomeDB), and is expressed in K562 cells. Thus, CUX1 represents the most likely candidate whose loss of binding in the presence of the alternative allele (A) of rs7255545 could result in increased gene expression. Together with EMSA data, these results suggest that haplotype-specific regulation is mediated by allele-dependent TF binding.

## Discussion

Despite vaccines and treatments, SARS-CoV-2 infection can still lead to severe clinical outcomes and death. Identifying genetic factors that influence susceptibility to infection and disease severity is essential to understanding pathophysiological mechanisms and finding new drug targets. GWAS and meta-analyses have revealed human genetic factors associated with the risk and severity of COVID-19, but the causal genes and variants remain unclear. Identifying these causal genes and variants remain a major challenge, especially since many of the variants are in non-coding regions, which complicates functional interpretation. While few studies have investigated their regulatory functions through bioinformatics approaches, using eQTLs and sQTLs databases, none have provided experimental validation to clearly identify causal variants.

In this study, we combined bioinformatics and experimental approaches and identified two novel causal variants that increase *DPP9* expression in lung and myeloid cells, thereby increasing the risk of severe COVID-19. We suggest that these variants can act through allele-specific binding of TFs in A549 and K562 cells. Our findings support the key role of *DPP9* in SARS-CoV2 infection and its potential as a therapeutic target for preventing severe COVID-19.

We selected the 13 lead SNPs associated with COVID-19 phenotypes [9] and identified 565 variants in LD with these SNPs. These were prioritized based on their potential functional relevance using bioinformatics tools and epigenomic data. To further investigate the genetic regulation of the ten top-scoring variants, we performed functional analyses in three relevant cell lines. Our results showed that haplotype of the alternative alleles of two *DPP9* variants (rs2277732 and rs7255545) increase the risk of severe COVID-19 and enhance gene reporter activity with and without stimulations, thereby suggesting that increased *DPP9* expression confers disease susceptibility. Haplotype analysis further strengthened this association with severe COVID-19, compared to single-variant analyses, supporting their combined functional effect.

Interestingly, the two index variants rs2277732 and rs7255545 were strongly associated with all three phenotypes studied in the COVID-19 HGI consortium [9], namely COVID-19 susceptibility, hospitalized COVID-19 cases, and very severe COVID19 (*P* = 7.3 × 10^–22^, 1.6 × 10^–47^, 7.3 × 10^–36^, for rs2277732, and* P* = 1.8 × 10^–7^, 1.4 × 10^–12^, 2.8 × 10^–11^, for rs7255545, for each of the three phenotypes, respectively). More generally, the three outcomes show extremely high genetic correlation (> 0.85 across all pairs of phenotypes) with most variants displaying concordant association across outcomes, although at different magnitudes. Although we were unable to investigate haplotype effects on all three phenotypes, as only hospitalized COVID-19 cases are available in the individual-level data from EpiGenCOV, one may expect a trend similar to the one observed for single variants. Our findings therefore provide the first functional evidence implicating both SNPs, rs2277732 and rs7255545, in COVID-19 susceptibility and severity. These variants have not previously been associated with other phenotypes, with the exception of a modest association between rs2277732 and idiopathic pulmonary fibrosis (*P* = 0.003), reported in the Global Biobank Meta-Analysis Initiative (GBMI) [36]. According to the GWAS catalogue [37], additional genome-wide significant associations have been reported for *DPP9* at the gene level, with traits such as body height, myeloid-derived growth factor levels, idiopathic pulmonary fibrosis, and interstitial lung disease. However, it should be noted that one of the limitations of GWAS is that they cannot identify rare mutations. We therefore cannot rule out the possibility that additional mutations in this locus may also contribute to severe COVID-19 susceptibility.

Our findings are consistent with studies reporting higher *DPP9* mRNA levels in the blood of COVID-19 patients [38] increasing with disease severity [39]. The association between severe disease and *DPP9* variants (rs2109069 and rs12610495), which are in LD with our candidate variants, has been replicated in multiple studies [40–42]. However, eQTL data from GTEx show that the risk alleles of these variants are paradoxically associated with reduced *DPP9* expression in the lungs. In our analysis, neither rs12610495 nor rs2109069 was prioritized by the IW scoring tool, and both lacked characteristics of regulatory function. This highlights the limits of eQTLs for functional characterization, as they only reflect the correlation of individual SNPs and gene expression, without demonstrating a functional effect. Unfortunately, we were unable to provide direct evidence linking the risk haplotype to increased *DPP9* expression in vivo. This requires modifying the alleles of the two regulatory variants, which are approximately 1,000 bp apart, by genome editing without introducing additional changes, which, is technically challenging.

GWASs reveal an overlap between genetic signals for COVID-19 severity and those for lung diseases, suggesting that *DPP9* may have a common role in their pathogenesis. This is consistent with epidemiological data associating pre-existing lung conditions with more severe COVID-19 outcomes [43, 44], and with the fact that respiratory failure remains a major cause of death among hospitalized COVID-19 patients [45, 46]. Pulmonary fibrosis is also a hallmark of severe COVID-19 [47], consistent with findings that the rs12610495 variant, associated with COVID-19 [48], also increases the risk of idiopathic pulmonary fibrosis (IPF) in independent studies [49–51]. In addition, higher *DPP9* expression levels have been observed in lung biopsies from IPF patients, but neither rs12610495 nor rs2109069 was found to influence *DPP9* expression [49], supporting that additional regulatory variants are likely to play a role in severe disease. Instead, we propose that the combination of alternative alleles of the rs2277732 and rs7255545, which increase gene expression in the lung and immune cells, may contribute to severe COVID-19, although the underlying mechanism remains unclear. Recent studies suggest that the role of DPP9 might extend beyond a simple increase in gene expression and involves more complex regulatory mechanisms, including the control of alternative splicing and inflammasome activation [52, 53]. In this context, the increased expression observed for the risk haplotype may have functional consequences not only at the quantitative level, but also by altering the balance of DPP9 isoforms or modulating its regulatory role in inflammatory pathways. Such mechanisms could contribute to disease pathogenesis by influencing the inflammatory response rather than through a strictly linear effect of expression levels.

DPP9 has recently been identified as a repressor of NLRP1 inflammasome activation [54]. NLRP1 is a highly expressed gene in epithelial barrier tissues, which initiates the innate immune response of the host. Notably, human NLRP1 can detect double-stranded RNA (dsRNA) and activate an inflammasome complex in response to dsRNA detection [55], suggesting that dsRNA replication intermediates of SARS-CoV-2 [56] could activate NLRP1, triggering an inflammatory cascade through inflammasome activation. While inflammasome activation can defend against pathogens, excessive activation may result in tissue damage and unrestrained immune cell recruitment. We propose that elevated DPP9 levels in severe COVID-19 may suppress inflammation [38, 39] by inhibiting NLRP1, preventing downstream inflammasome activation [57]. Thus, DPP9 might act as an intracellular checkpoint, limiting pyroptosis and excessive immune activation. However, overexpression of DPP9 in carriers of the risk haplotype could impair the innate immune response’s ability to restrict viral replication. This might help the virus evade immune assault at early stages of infection and eventually lead to the release of large quantities of cytokine from dying monocytes at later stages, which would promote hyperinflammation and a cytokine storm in patients [58].

Conversely, DPP9 also contributes to inflammation by promoting monocyte and macrophage activation. Studies indicate that DPP9 gene silencing in human monocytes [59] or inhibition of its activity in macrophages leads to impaired activation and reduced secretion of the proinflammatory cytokine IL-6 [60]. In severe COVID-19, monocytes and macrophages are likely major sources of the excessive proinflammatory mediators, including TNF-α and IL-6 in the respiratory tract [61]. IL-6 levels have also been associated with mortality, admission to intensive care and hospitalization, representing a poor prognostic factor for COVID-19 [62]. Recent reports have suggested that cytokine profiles of patients with severe COVID-19 show similarities to cytokine release syndromes, such as macrophage activation syndromes [61, 63, 64] and that monocytes and macrophages extensively accumulate in the lungs [65]. This feedback loop drives hyperinflammation and severe lung damage. Consistently, elevated *DPP9* levels have also been associated with fibrosis in renal tubular epithelial cells [66], and in inflamed lung tissues [67]. In addition, while DPP9 enzymatic activity is increased in experimental rat asthma, the mouse model of airway inflammation revealed a protective role for NLRP1 inflammasome activation by blocking DPP9 [68]. Furthermore, it was recently shown that DPP9 is also expressed in epithelial cells alters cell adhesion in human embryonic kidney cells [69] suggesting that pulmonary fibrosis may also arise from perturbation in cell–cell adhesion or cytoskeletal integrity, which may be unable to withstand the mechanical stress of stretching within the lung. Finally, it is possible that the increased regulatory activity associated with the risk haplotype at the *DPP9*locus may influence additional pathways relevant to COVID-19 pathophysiology, including coagulation processes. Indeed, human induced pluripotent stem cells stimulated with SARS-CoV-2 have shown significant upregulation of fibrinogen genes (FGA and FGG) [70], which have been associated with severe COVID-19 [71]. However, a direct link between DPP9 and specific coagulation factors such as fibrinogen has not yet been established and remains speculative. Further studies will be required to clarify whether DPP9 contributes to the thrombotic and pulmonary manifestations observed in severe COVID-19. Thus, DPP9 may play a key role in mediating lung damage observed in severe cases of COVID-19 and can serve as a biomarker for disease severity.

## Conclusions

We identified two causal variants whose haplotype of alternative alleles increases both DPP9 levels and the risk of severe COVID-19. However, the role of DPP9 in SARS-CoV 2 infections appears dual: it can suppress inflammation by inhibiting NLRP1, but its overexpression may trigger lung hyperinflammation by activating monocytes and macrophages and disrupting cellular integrity. These effects can lead to cytokines storms and lung damage, responsible for respiratory failure.

## Supplementary Information

Additional file 1: Supplementary Table S1-S6

Additional file 2: Supplementary Fig S1 and S2

## Data Availability

This study uses existing de-identified individual-level data from two existing cohorts (French COVID and Milieu Interieur) and did not produce any new data. The data were accessed using the project submission system specific to each cohort. Sample request form for Milieu Interieur is available from https://www.milieuinterieur.fr/en/contact-us/access-to-mi-data-and-samples/. Data for French COVID are available upon request with synopsis by contacting Professor Jade GHOSN via jade.ghosn@aphp.fr. This study also uses publicly available summary statistics from the COVID-19 Host Genome Initiative. They are available for download at https://www.covid19hg.org/results/r7/. All other data supporting the findings of this study are included in the article and in the Supplementary Information files. The raw numbers for charts and graphs are available in the Source Data file. Requests for materials and correspondence should be addressed to Sandrine Marquet.

## Abbreviations

SARS-CoV-2: Severe Acute Respiratory Syndrome Coronavirus 2
GWAS: Genome-wide association studies
*DPP9: *: Dipeptidyl peptidase 9
SNP: Single nucleotide polymorphism
CHGE: COVID Human Genetic Effort
GBMI: Global Biobank Meta-Analysis Initiative
IFNα: Interferon alpha
IFNγ: Interferon gamma
TNF α: Tumor necrosis factor alpha
HGI: Host Genetics Initiative
LD: Linkage disequilibrium
eQTL: Expression quantitative trait loci
sQTL: Splicing quantitative trait loci
TFBS: Transcription factor binding sites
TF: Transcription factors
Ref: Reference
Alt: Alternative
mRNA: Messenger RNA
EMSA: Electrophoretic mobility shift assay
NE: Nuclear extract
IPF: Idiopathic pulmonary fibrosis
dsRNA: Double stranded RNA
IL-6: Interleukin 6
IW: Integrative Weighted
MAF: Minor allele frequency
