# Supplementary material for: Identification and functional characterization of regulatory variants in DPP9 associated with COVID-19 severity
Source: Genome Med. 2026 Jul 2;18:138. doi: 10.1186/s13073-026-01703-0 (PMC13602522; doi:10.1186/s13073-026-01703-0)
Supplement: Supplementary file 1 [file 13073_2026_1703_MOESM2_ESM.pdf]

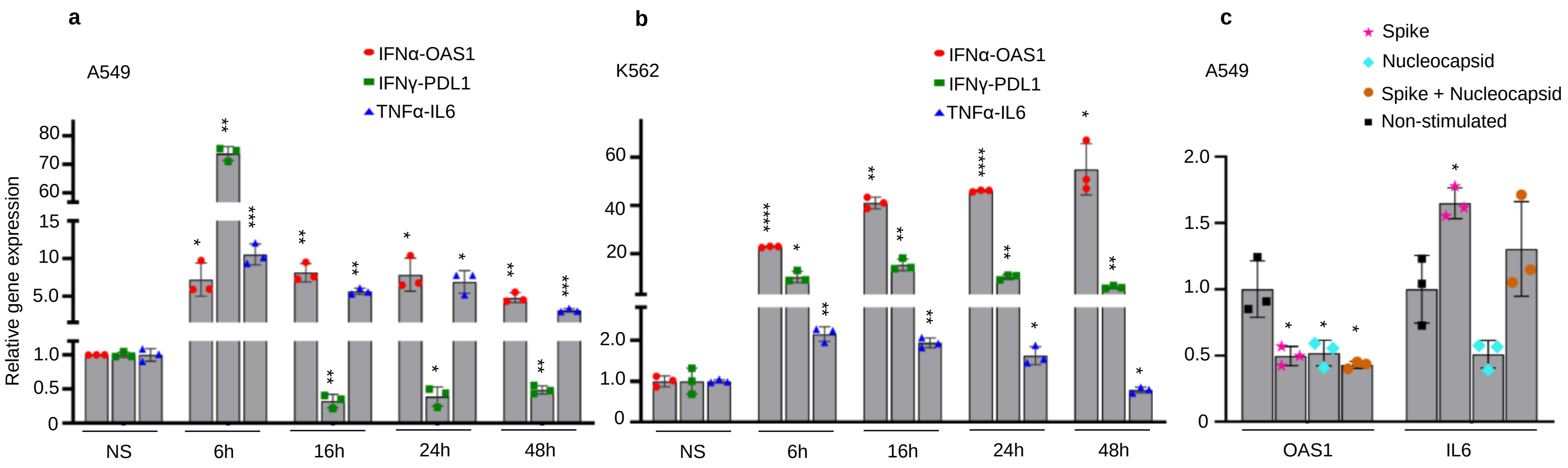


Fig S1: Quantification of control genes for cytokine and recombinant SARS-CoV-2 protein stimulation efficiency.

a mRNA analysis of OAS1, PDL1 and IL6 as controls for IFNα, IFNγ and TNFα stimulations in A549 cells. b mRNA analysis of OAS1, PDL1 and IL6 as controls for IFNα, IFNγ and TNFα stimulations in K562 cells. c mRNA analysis of IL6 and OAS1 as controls for recombinant human coronavirus SARS-Cov-2 Spike glycoprotein RBD and Nucleocapsid protein stimulations in A549 cells. Data shows relative gene expressions normalized to GAPDH housekeeping gene as mean values ± SD of n = 3 independent replicates. Significance was determined using a two tailed Student t test and P-values are represented as asterisks (* $P < 0.05$; ** $P < 0.01$; *** $P < 0.0001$; **** $P < 0.00001$). Source data and exact P-values of statistical tests are provided in Source Data file.

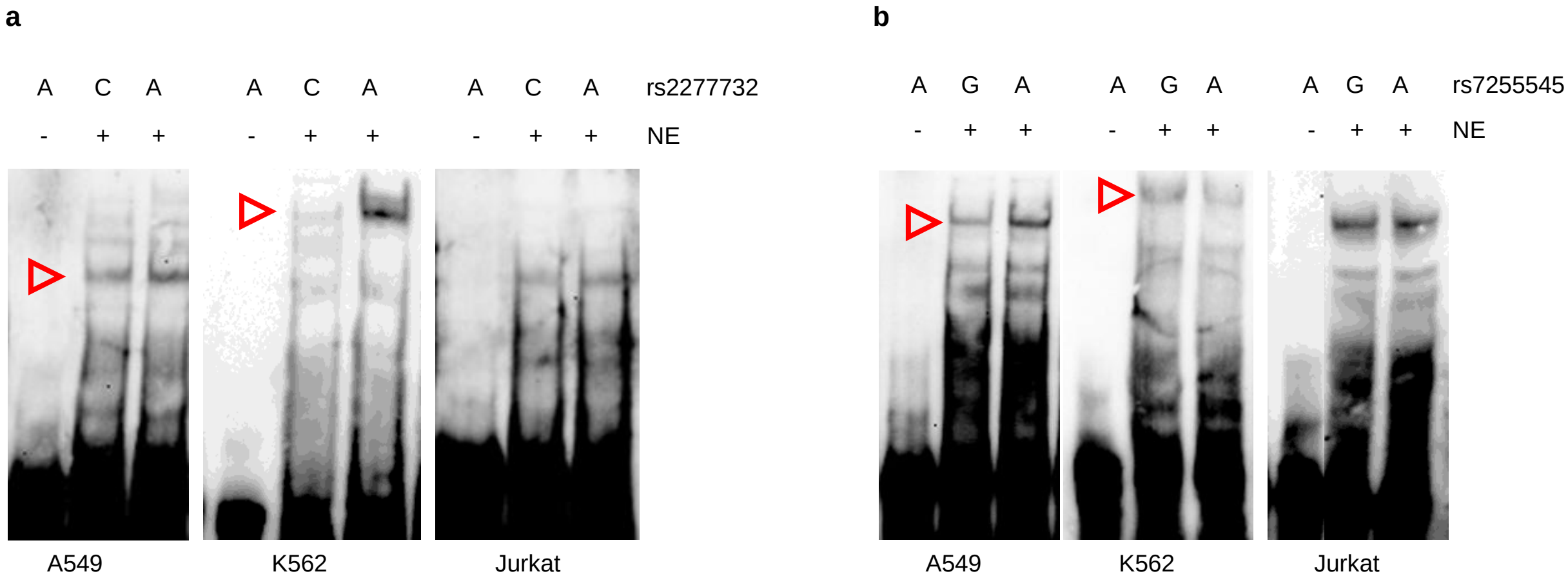


Fig S2: DPP9 variants alter the binding of TFs in a cell-type specific manner.

a, b EMSA were performed in A549, K562, and Jurkat cell lines to assess the effect of rs2277732 (a) and rs7255545 (b) alleles on TF binding using corresponding biotinylated probes. EMSA experiments were independently repeated twice, yielding consistent results.
